# Burnout and perceived stress as predictors of quality of life in nurses

**DOI:** 10.15649/cuidarte.5113

**Published:** 2025-10-17

**Authors:** Alexandra Estrella Buitron Saavedra, Alessandra Cricel Quispe Caballero, Jonatan Baños Chaparro

**Affiliations:** 1 Universidad Científica del Sur, School of Psychology, Lima, Peru. E-mail: 100048167@cientifica.edu.pe Universidad Científica del Sur Lima Perú 100048167@cientifica.edu.pe; 2 Universidad Científica del Sur, School of Psychology, Lima, Peru. E-mail: 100037941@cientifica.edu.pe Universidad Científica del Sur Lima Perú 100037941@cientifica.edu.pe; 3 Universidad Privada Norbert Wiener, Vice- Rectorate for Research, Lima, Peru. E-mail: jonatan.banos@uwiener.edu.pe Universidad Privada Norbert Wiener Lima Perú jonatan.banos@uwiener.edu.pe

**Keywords:** Burnout, Professional, Stress, Psychological, Quality of Life, Primary Prevention, Mental Health, Agotamiento Profesional, Estrés Psicológico, Calidad de Vida, Prevención Primaria, Salud Mental, Esgotamento Profissional, Estresse Psicológico, Qualidade de Vida, Prevenção Primária, Saúde Mental

## Abstract

**Introduction:**

Burnout and perceived stress are prominent psychological problems manifested by nursing staff and substantially affect the quality of life and performance in daily activities of licensed nurses and nursing technicians.

**Objective:**

To analyze the influence of burnout and perceived stress on the quality of life of nursing personnel.

**Materials and Methods:**

Predictive, comparative, cross- sectional, quantitative study. A total of 450 participants (42.89% registered nurses and 57.11% nursing technicians) from a public military hospital completed psychological instruments and were recruited through non-probability convenience sampling. Structural equation modeling and Bayesian analysis were performed.

**Results:**

The model demonstrated adequate fit, and negative stress (β = -0.59, p = 0.00) was identified as a statistically significant negative predictor of mental health. In addition, registered nurses reported higher levels of negative stress, depersonalization, and emotional exhaustion compared to nursing technicians.

**Discussion:**

Negative stress is a determinant of mental health in nurses, a finding consistent with empirical evidence in highly demanding work environments such as hospitals. The greater symptoms observed in registered nurses can be explained by their greater responsibility for patient care and the institution.

**Conclusion:**

Negative stress is a significant predictor of mental health in nursing personnel, and registered nurses reported higher levels of negative stress, depersonalization, and emotional exhaustion. Effective prevention strategies are needed to manage stress and burnout in clinical contexts.

## Resumen

**Introducción:**

El burnout y el estrés percibido son problemas psicológicos prominentes manifestados por el personal de enfermería y afectan de manera sustancial la calidad de vida y el desempeño en las actividades cotidianas de enfermeros titulados y técnicos en enfermería.

**Objetivo:**

Analizar la influencia del burnout y el estrés percibido en la calidad de vida del personal de enfermería.

**Materiales y métodos:**

Estudio predictivo, comparativo, transversal y con enfoque cuantitativo. Participaron 450 enfermeros (42,89%) y técnicos en enfermería (57,11%) de un hospital público militar, quienes respondieron instrumentos psicológicos y fueron seleccionados mediante muestreo no probabilístico por conveniencia. Se aplicaron modelos de ecuaciones estructurales y análisis bayesiano.

**Resultados:**

El modelo presentó un ajuste adecuado y el estrés negativo (β = -0,59; p = 0,00) se identificó como un predictor negativo y estadísticamente significativo de la salud mental. Además, los enfermeros titulados, en comparación con los técnicos en enfermería, presentaron mayor estrés negativo, despersonalización y agotamiento emocional.

**Discusión:**

El estrés negativo es un determinante de la salud mental en enfermeros, lo que coincide con la evidencia empírica en entornos laborales de alta demanda como los hospitales. La mayor sintomatología en enfermeros titulados puede explicarse por la mayor responsabilidad frente al paciente y la institución.

**Conclusiones:**

El estrés negativo es un predictor de la salud mental en el personal de enfermería, y los enfermeros titulados reportan mayores niveles de estrés negativo, despersonalización y agotamiento emocional. Es necesario implementar estrategias efectivas de prevención para el manejo del estrés y el burnout en contextos clínicos.

## Resumo

**Introdução:**

Burnout e estresse percebido são problemas psicológicos proeminentes manifestados pela equipe de enfermagem e afetam substancialmente a qualidade de vida e o desempenho nas atividades diárias de enfermeiros e técnicos de enfermagem.

**Objetivo:**

Analisar a influência do burnout e do estresse percebido na qualidade de vida de profissionais de enfermagem.

**Materiais e métodos:**

Pesquisa preditiva, comparativa, transversal e com abordagem quantitativa. Participaram 450 enfermeiros (42,89%) e técnicos de enfermagem (57,11%) de um hospital militar público, que responderam a instrumentos psicológicos e foram selecionados por amostragem não probabilística por conveniência. Foram realizadas modelagem de equações estruturais e análise bayesiana.

**Resultados:**

O modelo apresentou ajuste adequado e estresse negativo (β = -0,59, p = 0,00) e foi considerado um preditor negativo estatisticamente significativo da saúde mental. Além disso, enfermeiros, em comparação aos técnicos de enfermagem, apresentaram maior estresse negativo, despersonalização e exaustão emocional.

**Discussão:**

O estresse negativo é um determinante da saúde mental em enfermeiros e este achado é consistente com evidências empíricas em ambientes de trabalho de alta demanda, como hospitais. A maior sintomatologia em enfermeiros licenciados pode ser explicada pela maior responsabilidade em relação ao paciente e à instituição.

**Conclusões:**

O estresse negativo é um preditor da saúde mental em profissionais de enfermagem e enfermeiros licenciados relatam maior estresse negativo, despersonalização e exaustão emocional. É necessário implementar estratégias eficazes de prevenção para o gerenciamento do estresse e do burnout em contextos clínicos.

## Introduction

Nursing personnel play a fundamental role in community healthcare. In their daily activities, registered nurses and nursing technicians are exposed to stressors that can affect their psychological and physical well-being, which negatively influence their quality of life^1^-^3^. Nursing practice in public entities, such as hospitals, is an exhausting labor, as the demands of the activities, patient pressure, rotating shift schedules, and conflictive work environments trigger psychological risk factors due to the demanding nature of the work^4^-^6^.

Several systematic reviews and meta-analyses report that healthcare personnel, especially nurses, are particularly vulnerable to various psychological problems, particularly burnout syndrome and perceived stress^7^-^9^. Particular features of burnout syndrome are emotional exhaustion, depersonalization, and reduced personal accomplishment in the workplace. Evidence among nursing personnel suggests that burnout syndrome affects professional functionality and capacity, as it can be directly related to low motivation, feelings of being underappreciated, uncontrolled procrastination, feelings of inadequacy, negative attitudes, and poor stress management. All this leads to psychological fatigue and low job satisfaction, since professionals may feel psychologically incapable of performing their work duties, which affects their quality of life^10^.

Perceived stress, in turn, is defined as a state of repeated tension caused by a situation appraised as dangerous, because, when performing a self-assessment, the individual believes that they lack the necessary resources to face that circumstance^11^,^12^. In nursing practice, stress is a health risk factor, producing various physical, emotional, and social issues that directly affect nurses' quality of life and negatively impact patient care^10^.

The interaction between burnout syndrome and perceived stress has an adverse effect on the quality of life of nursing personnel, which is considered a person's overall well-being in terms of physical, psychological, economic, social, and environmental health^10^,^13^,^14^. Evidence suggests that the coexistence of burnout and stress is closely related to psychological and physical problems, which negatively affect nursing personnel's quality of life^15^. In fact, several studies indicate that prolonged stress and burnout have a significant impact on nurses' job performance, as they affect concentration, commitment, productivity, and sense of accomplishment^6^.

It is relevant to note that, although research on this problem exists among Peruvian nurses, most studies have focused on registered nurses rather than nursing technicians. Nursing technicians constitute a fundamental part of the health team and face similar or greater challenges, as they are often engaged in prolonged contact with patients through immediate care. In contrast, registered nurses assume leadership, decision-making, and health management functions in their area of work^16^-^18^. It is widely recognized that nursing technicians experience higher levels of burnout and stress. However, other studies suggest that there are no differences, since both groups perform demanding and exhausting tasks. In many cases, work is done in teams, which means that both professional categories share the same tasks^19^,^20^.

Given the importance of mental health in health professionals, it is necessary to understand the psychological risk factors and the differences or similarities between registered nurses and nursing technicians to improve and develop effective interventions that promote the physical and mental well-being of nursing personnel, ultimately contributing to a better quality of care for the community. For this reason, the study aimed to analyze the influence of burnout and perceived stress on the quality of life of nursing personnel.

## Materials and Methods

**Design**

This was a cross-sectional quantitative study. A comparative design was chosen, using an associative predictive strategy with latent and observable variables^21^.

**Participants**

A total of 450 nurses and nursing technicians from the Hospital Central FAP Comandante FAP Médico Juan Benavides Dorich participated in the study. Of the participants, 193 (42.89%) were nurses, 257 (57.11%) were nursing technicians, 394 (87.56%) were women, and 56 (12.44%) were men. Non- probability convenience sampling was applied, and the following inclusion criteria were used: a) age between 20 and 65 years, b) either sex, c) being a registered nurse or nursing technician, and d) working at the Hospital Central FAP. The established exclusion criteria were: a) absence on the day of data collection, b) unwillingness to participate, and c) not signing the informed consent form.

**Instruments**

**Maslach Burnout Inventory (MBI-HSS).** This instrument consists of 15 questions grouped into three dimensions: emotional exhaustion, depersonalization, and personal accomplishment. Responses are rated on a Likert scale ranging from 0 to 6, reflecting the frequency of the different situations described in the survey experienced by the individual. Its purpose is to evaluate the presence or absence of burnout across the three dimensions. The version adapted to the Peruvian context was used, with adequate reliability for each dimension: emotional exhaustion (α = 0.73), depersonalization (α = 0.69), and personal accomplishment (α = 0.68)^22^.

**Perceived Stress Scale (PSS-10).** The PSS-10 comprises 10 items grouped into two dimensions: positive stress and negative stress. Responses are rated on a Likert scale ranging from never to always. The purpose of the instrument is to measure the level at which individuals perceive the different scenarios in their lives as stressful. The Peruvian adaptation was applied, which has an adequate reliability for each dimension: positive stress (α = 0.81, ω = 0.85) and negative stress (α = 0.68, ω = 0.76)^23^.

**12-Item Short-Form Health Survey (SF-12).** The SF-12 is a two-dimensional scale that assesses an individual's perceived health in both mental and physical domains. It has a Likert-type and, in some cases, dichotomous response options. The version adapted to the Peruvian context was used, which showed adequate reliability (α = 0.81, ω = 0.84)^24^.

**Data Analysis**

The statistical analysis process was conducted in the open-access programs RStudio and JASP. In the first part, a descriptive analysis of the psychological variables was performed using the arithmetic mean, standard deviation, and Pearson's correlation matrix. For interpretation of correlations, the effect size was considered according to the following criteria: small = 0.10, medium = 0.20, large = 0.30, and very large = 0.40^25^.

In the second part, inferential regression analysis was conducted using structural equation modeling (SEM), which employed latent variables to estimate standardized regression parameters, assess statistical significance, and evaluate model fit indices. Given the nature of continuous variables, the robust maximum likelihood estimator (MLR) was applied, as it is recommended for data that do not meet multivariate normality assuptions^26^. Model evaluation was performed in two stages. The first stage corresponds to the global evaluation, which considered the comparative fit index (CFI > 0.90), the root mean square error of approximation (RMSEA < 0.08), and the standardized root mean square residual (SRMR < 0.08)^27^. If fit indices were not satisfactory, modification indices (>10) for the covariance of observed variable errors were examined. The second stage corresponds to local evaluation, in which standardized regression coefficients (β) and statistical significance (p < 0.05) were assessed.

Finally, in the third part, a comparative analysis was conducted based on the type of nursing personnel (registered nurses or nursing technicians). Population distribution was first tested using the Shapiro- Wilk (SW) test, which indicated non-normality of data distribution (SW < 0.05). Therefore, the Bayesian Mann-Whitney U test was applied to compare independent groups. Hypothesis testing employed the Bayes factor (BF) to estimate posterior distributions for the alternative hypothesis (BF10) and null hypothesis (BF01). The iterative BF procedure begins with the Cauchy prior distribution with a standard r = 0.70, and the model convergence fit was evaluated using the R-hat test (> 1)^28^,^29^. To strengthen the results, a robust analysis was performed with Cauchy priors set at r = 1 and r = 1.41^29^. To facilitate interpretation of BF values, Jeffreys' scale was followed, which classifies the evidence into anecdotal (BF0+ = 1-3), moderate (BF0+ = 3-10), strong (BF0+ = 10-30), and very strong (BF0+ = 30-100)^30^. Scientific evidence suggests that BF > 10 provides adequate support for interpretation. All data collected are available for free access and consultation on the Open Science Framework (OSF) repository^31^.

**Procedure**

Data was collected in person between June and December 2024 at the public military hospital, which is part of the Dirección de Redes Integradas de Salud (DIRIS Lima Centro). The hospital is classified as a Level III-1 facility, as it offers medical specialties and subspecialties.

Initially, the research proposal was submitted to the hospital's teaching and research office for review. Following approval by the hospital's research ethics committee, a formal letter was sent to the hospital director, who authorized implementation of the study. Data collection was conducted in three phases: the first phase included the entire outpatient service, the second phase covered hospitalization areas (internal medicine, surgery, pediatric, gynecology-obstetrics, mental health, communicable diseases, neurosurgery, trauma unit, neonatology, medical-surgical unit, geriatrics, and central sterile services), and the third phase focused on critical care units such as intensive care, intermediate care, and emergency services. In all three phases, the researchers were present to provide instructions and oversee the completion of the questionnaires. Important points were emphasized during the presentation of the research to the potential participants, including anonymity, data confidentiality, and voluntary participation. The requirement to complete the informed consent before filling out the questionnaires was informed. During the administration of the instruments, the researchers explained the order of completion, starting with the sociodemographic data form and then proceeding to the study instruments. In relation to this, the importance of answering honestly was stressed, and participants were reminded of their right to withdraw from the study whenever they wished.

**Ethical considerations**

The present research was approved on April 26, 2024, by the ethics and research committee of the Universidad Científica del Sur (registration code PRE-18-2024-00031. The study adhered to the ethical principles of the American Psychological Association^32^, ensuring the responsible and confidential treatment of data for research purposes. Furthermore, each participant signed an informed consent form and was informed of their right to withdraw from the study at any time.

## Results

**Descriptive Analysis**

**Figure 1:**
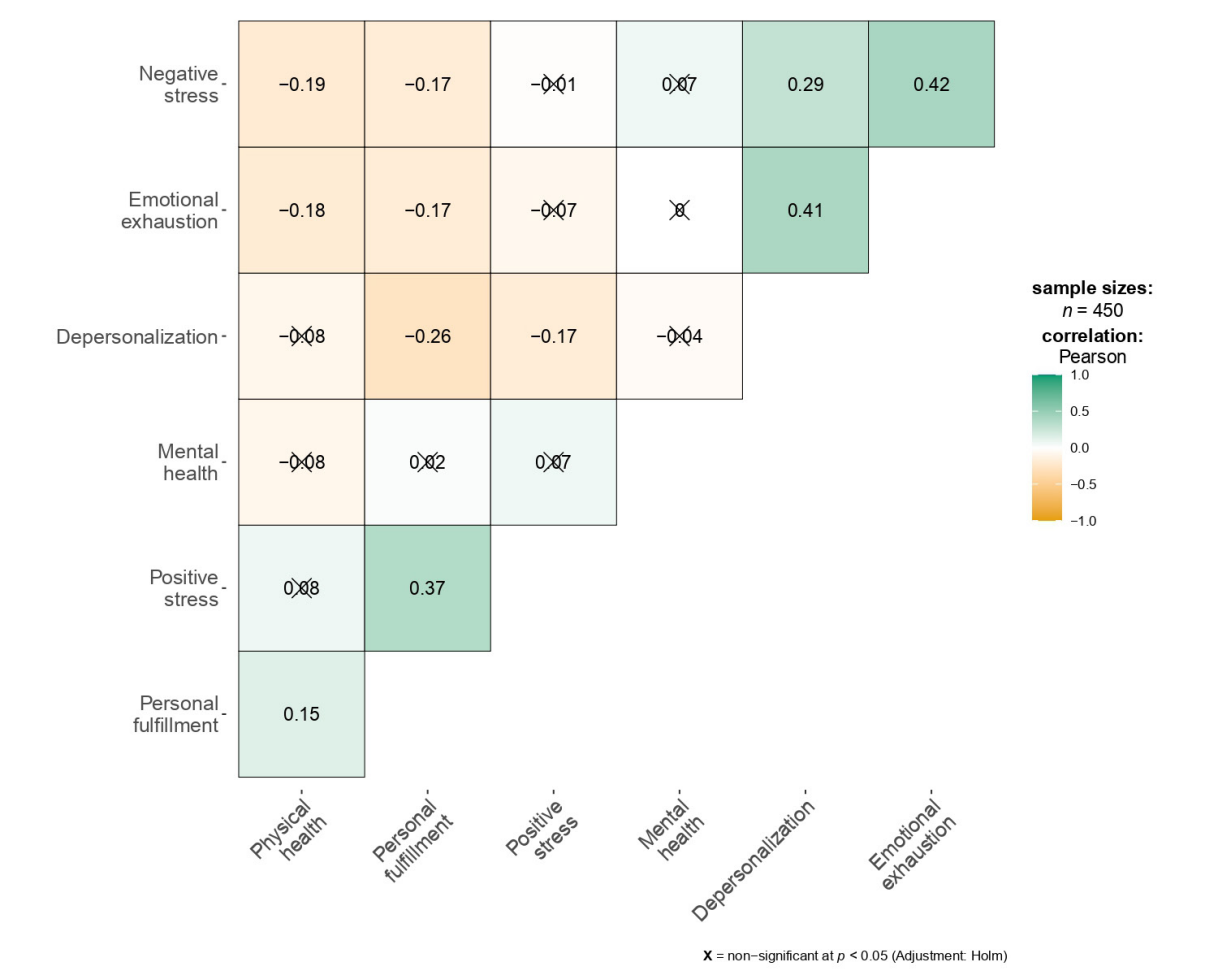
Correlation matrix among burnout, perceived stress, and quality of life in Peruvian nurses

*Note: X = non-significant at p<0.05(Adjustment Homl)*

The highest arithmetic mean (M) was observed for personal accomplishment (M = 27.19), while the lowest was for depersonalization (M = 2.09). For standard deviation (SD), the highest value corresponded to personal accomplishment (SD = 5.98) and the lowest to physical health (SD = 1.67). Regarding the correlation matrix among the psychological variables, significant and non-significant correlations were found, with different effect sizes ranging from small to very large (Figure 1).

**Predictive Analysis**

**Figure 2:**
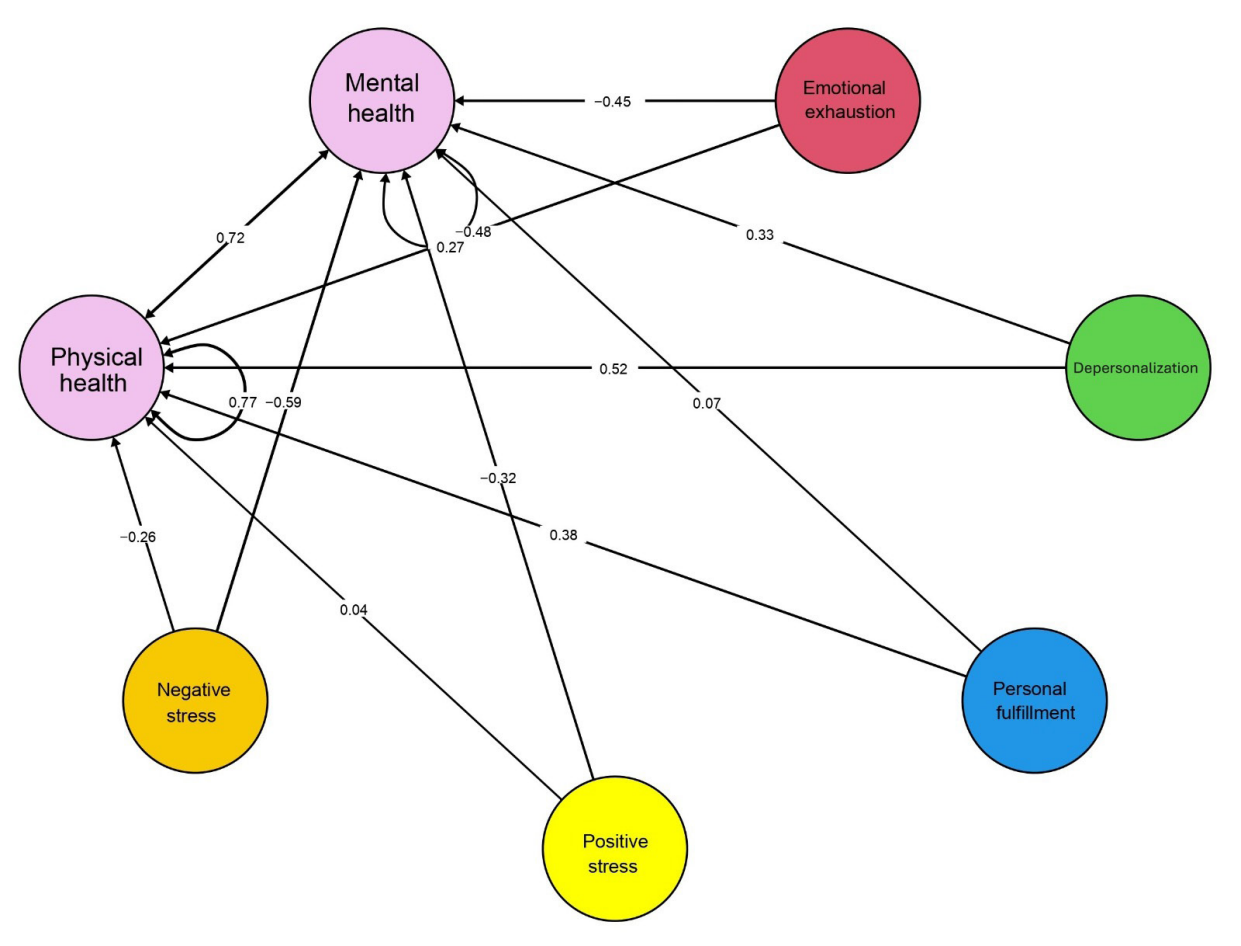
Regression model between burnout, perceived stress, and quality of life in Peruvian nurses

The structural equation model demonstrated acceptable fit after allowing covariance between two residual errors: CFI = 0.90, RMSEA = 0.03 [90% IC: 0.02-0.03], and SRMR = 0.05. For mental health, only negative stress (β = -0.59, p = 0.00) was found to be a statistically significant negative predictor. Emotional exhaustion (β = -0.45, p = 0.09) and positive stress (β = -0.32, p = 0.24) were non-significant negative predictors of mental health, while depersonalization (β = 0.33, p = 0.39) and personal accomplishment (β = 0.07, p = 0.64) were non-significant positive predictors (Figure 2).

For physical health, no statistically significant regression model was identified. However, emotional exhaustion (β = -0.48, p = 0.21) and negative stress (β = -0.26, p = 0.22) were identified as non-significant negative predictors, whereas depersonalization (β = 0.52, p = 0.37), personal accomplishment (β = 0.38, p = 0.09), and positive stress (β = 0.04, p = 0.80) appeared as non-significant positive predictors (Figure 2).

**Comparative Analysis**

Model convergence was acceptable according to the R-hat statistic (range = 1.00-1.01). Table 1 presents strong evidence supporting the null hypothesis for negative stress (BF01 = 17.41), depersonalization (BF01 = 12.10), and emotional exhaustion (BF01 = 11.30).

In particular, negative stress scores were slightly higher in registered nurses (M = 6.07) than in nursing technicians (M = 5.95). Similarly, depersonalization was also higher among registered nurses (M = 2.17) compared to nursing technicians (M = 2.03). Emotional exhaustion scores were greater in registered nurses (M = 6.46) than in nursing technicians (M = 6.05).

**Table 1:** Bayes factor for burnout, perceived stress, and quality of life in registered nurses and nursing technicians

	Cauchy prior distribution					
	r = 0.70		r = 1.00		r = 1.41	
	BF10	BF01	BF10	BF01	BF10	BF01
Emotional exhaustion	0.17	5.79	0.12	7.98	0.08	11.30
Depersonalization	0.16	6.22	0.11	9.07	0.08	12.10
Personal accomplishment	0.29	3.40	0.27	3.68	0.20	4.81
Positive stress	0.24	4.06	0.19	5.02	0.14	6.93
Negative stress	0.10	9.13	0.08	12.03	0.05	17.41
Physical health	0.88	1.13	0.35	2.83	0.53	1.86
Mental health	0.64	1.55	0.33	3.01	0.32	3.08

r: Cauchy prior distribution under H1.

## Discussion

The scientific literature clearly establishes the challenges and work demands faced by nursing personnel in public and private health services. Burnout syndrome and perceived stress are prominent psychological problems reported by nursing personnel. They are known to substantially affect the quality of life and performance in daily activities of registered nurses and nursing technicians. Therefore, identifying the impact of these psychological conditions on the quality of life of registered nurses and nursing technicians is essential for promoting the improvement and well-being of nursing personnel.

It was identified that only negative stress was a statistically significant negative predictor of mental health in nursing personnel. This finding is consistent with empirical evidence showing that chronic stress, especially in highly demanding work settings such as hospitals, has adverse effects on the psychological well-being of nursing personnel^33^,^34^. A plausible explanation is the constant exposure to emergencies, excessive workload, and emotional strain of caring for critical patients, all of which can contribute to a sustained and prolonged state of negative stress that affects mental health^35^. Similarly, some studies report that this problem may be exacerbated by the lack of resources and coping skills among nurses, combined with the lack of institutional support and limited self-care information, which can hinder effective stress management in healthcare workers^36^.

In addition, emotional exhaustion and positive stress were found to be negative predictors of mental health, while depersonalization and personal accomplishment were positive predictors; however, none reached statistical significance. Despite the absence of statistical significance, the prediction coefficients are consistent with the scientific literature. It has been documented that emotional exhaustion is an important predictor of mental health among health professionals^37^. The negative predictive association between positive stress and mental health could be explained by the coping strategies used by registered nurses and nursing technicians to overcome problems and adapt to changes, which are often influenced by personality factors and emotional management^38^. Conversely, the positive predictive values of depersonalization and personal accomplishment suggest a self- protective role in certain contexts to buffer emotional exhaustion caused by workload and enhance the psychological well-being of healthcare workers^39^. However, the lack of statistical significance of these predictors prevents drawing firm conclusions, which may be attributable to methodological or contextual aspects.

Likewise, no statistically significant predictions were identified for physical health. Nevertheless, it was observed that emotional exhaustion and negative stress tend to predict lower physical health, while depersonalization and personal accomplishment tend to predict higher physical health. Previous studies have considered negative stress as a relevant predictor of physical health in registered nurses. The negative predictive association between negative stress and physical health could be explained by lifestyle factors, self-care, health monitoring, physical activity, good eating habits, and avoidance of alcohol or drug use^40^. Similarly, emotional exhaustion is commonly reported by both registered nurses and nursing technicians, but its impact can be mitigated through healthy lifestyle practices^41^,^42^. By contrast, positive predictive associations of depersonalization and personal accomplishment may occur in circumstances where staff exercise greater independence and autonomy in decision-making and demonstrate greater disengagement from patients^43^.

In relation to the comparative analysis between registered nurses and nursing technicians, higher levels of negative stress, depersonalization, and emotional exhaustion were observed in registered nurses. Prior research suggests that registered nurses, due to their greater responsibilities in patient care, supervision of staff, and decision-making, are exposed to higher work pressure and may report greater stress than nursing technicians, whose tasks are more practical, involve less responsibility, and are carried out under the supervision of registered nurses; however, other studies report the opposite^44^,^45^. Depersonalization and emotional exhaustion among registered nurses may also be explained by factors related to emotional burden from demanding work, such as caring for severely ill patients in critical units, uncertainty regarding treatments, hierarchical conflicts, insufficient preparation, inadequate professional support, and conflicts with colleagues^46^,^47^.

On the other hand, the absence of differences in personal accomplishment, positive stress, physical health, and mental health appears to be independent of educational background and assigned work activities; both groups of registered nurses and nursing technicians perceived these aspects similarly. Personal accomplishment could be influenced by individual factors such as self-esteem, personal satisfaction, motivation, and resilience in the face of adversity, rather than by educational background or job functions^36^.

Likewise, the absence of differences in positive stress, physical health, and mental health between registered nurses and nursing technicians indicates how personal variables play a fundamental role in coping with stress. Lifestyle choices and problem-solving strategies they use in different aspects of their lives also play an important role in managing stress^48^,^49^.

The research findings have significant implications for theory and professional practice. From a theoretical standpoint, negative stress is a determinant of mental health among nursing personnel. This result is consistent with demand-control models of stress, which describe that an overwhelming appraisal of work demands and inadequate perceived resources contribute to professional burnout, affecting and underscoring the importance of mental health in healthcare workers. Furthermore, the higher levels of negative stress, depersonalization, and emotional exhaustion observed among registered nurses reinforce the existing literature, which indicates that this group bears a greater responsibility and emotional burden compared to nursing technicians. From a professional practice perspective, the findings suggest the need for interventions at both individual and organizational levels to reduce negative stress and mitigate its impact on mental health. Registered nurses, who are more exposed to negative stress, depersonalization, and emotional exhaustion, may benefit from preventive programs that focus on coping strategies, conflict resolution skills, relaxation techniques, and other psychological resources to change their perceptions of emotional burden and promote better adaptation to work demands.

The strengths of this study lie in its statistical approach, including the use of Bayesian analysis and structural equation modeling, as well as the balanced participation of registered nurses and nursing technicians. However, it is essential to mention some limitations despite the relevance of the study. First, the sample was limited to a specific site, limiting the generalizability of the findings to other healthcare centers. Second, the use of non-probability convenience sampling makes it impossible to extend the results to other healthcare professionals. Third, the data collection was cross-sectional, which prevented causal inferences; future studies should incorporate longitudinal designs to confirm these results. Fourth, the study sample was predominantly female, which may introduce gender bias; results could differ if this aspect is addressed. Finally, only the proposed psychological variables were included in the model, although other psychological and social factors can also influence the quality of life of nursing personnel.

## Conclusions

In conclusion, negative stress has a significant impact on the mental health of nursing personnel. Moreover, the registered nurses reported higher levels of negative stress, depersonalization, and emotional exhaustion compared to nursing technicians. These findings highlight the importance of mental health in clinical practice and the need to implement effective prevention strategies for stress management, burnout reduction, and the provision of substantial emotional support. Healthcare institutions should take steps to improve working conditions and provide adequate resources that promote the psychological well-being of all healthcare professionals.
